# Drivers of tropical forest loss between 2008 and 2019

**DOI:** 10.1038/s41597-022-01227-3

**Published:** 2022-04-01

**Authors:** Juan Carlos Laso Bayas, Linda See, Ivelina Georgieva, Dmitry Schepaschenko, Olga Danylo, Martina Dürauer, Hedwig Bartl, Florian Hofhansl, Roman Zadorozhniuk, Maksym Burianchuk, Flavius Sirbu, Brigitte Magori, Kateryna Blyshchyk, Volodymyr Blyshchyk, Ahmed Harb Rabia, Chandra Kant Pawe, Yuan-Fong Su, Merajuddin Ahmed, Kripal Panging, Oleksandr Melnyk, Olesia Vasylyshyn, Roman Vasylyshyn, Andrii Bilous, Svitlana Bilous, Krishna Das, Reinhard Prestele, Ana Pérez-Hoyos, Khangsembou Bungnamei, Andrii Lashchenko, Maryna Lakyda, Ivan Lakyda, Oleksandr Serediuk, Galyna Domashovets, Yuriy Yurchuk, Michèle Koper, Steffen Fritz

**Affiliations:** 1 International Institute for Applied Systems Analysis (IIASA), Laxenburg, 2361 Austria; 2 National University of Life and Environmental Sciences of Ukraine (NULESU), Heroiv Oborony 15, Kyiv, 03041 Ukraine; 3 West University of Timisoara, Bulevardul Vasile Parvan 4, Timisoara, 300323 Romania; 4 Damanhour University, Faculty of Agriculture, Natural Resources & Agricultural Engineering Department, El-abaadya Campus, Damanhour, 22516 El-Behera, Egypt; 5 Pragjyotish College, Department of Geography, Guwahati, 09 Assam India; 6 National Taiwan Ocean University, Department of Harbor and River Engineering, No. 2, Beining Rd., Zhongzheng Dist., Keelung City, 202 Taiwan (R.O.C.); 7 Northeast centre for Equity Action on Integrated Development (NEAID), NEAID (Registered Office), G.R.C Road, Noonmati, Guwahati, Kamrup(M), Assam 781020 India; 8 Gauhati University, Department of Geography, Jalukbari, Guwahati, Assam 781014 India; 9 Karlsruhe Institute of Technology (KIT), Institute of Meteorology and Climate Research - Atmospheric Environmental Research (IMK-IFU), Kreuzeckbahnstraße 19, 82467 Garmisch-Partenkirchen, Germany; 10 Joint Research Center of the European Union (JRC), Via Enrico Fermi, 2749, I-21027 Ispra, Italy; 11 Guidehouse, Stadsplateau 15, 3521 Utrecht, AZ The Netherlands

**Keywords:** Environmental impact, Forestry, Agriculture, Natural hazards, Ecosystem services

## Abstract

During December 2020, a crowdsourcing campaign to understand what has been driving tropical forest loss during the past decade was undertaken. For 2 weeks, 58 participants from several countries reviewed almost 115 K unique locations in the tropics, identifying drivers of forest loss (derived from the Global Forest Watch map) between 2008 and 2019. Previous studies have produced global maps of drivers of forest loss, but the current campaign increased the resolution and the sample size across the tropics to provide a more accurate mapping of crucial factors leading to forest loss. The data were collected using the Geo-Wiki platform (www.geo-wiki.org) where the participants were asked to select the predominant and secondary forest loss drivers amongst a list of potential factors indicating evidence of visible human impact such as roads, trails, or buildings. The data described here are openly available and can be employed to produce updated maps of tropical drivers of forest loss, which in turn can be used to support policy makers in their decision-making and inform the public.

## Abstract (summary)

**Table:** 

Measurement(s)	drivers of tree loss
Technology Type(s)	visual inspection of very high resolution satellite imagery
Sample Characteristic - Organism	trees
Sample Characteristic - Environment	Forests
Sample Characteristic - Location	Tropics and subtropics

## Background & Summary

Reducing the rate of deforestation is a key global challenge for addressing climate change^1^, halting biodiversity loss^2^ and preserving crucial forest ecosystems services, such as carbon sequestration, timber production, and water retention^3^. The current rates of deforestation are estimated to be around 10 million ha per year, driven primarily by agricultural expansion^4^. However, the direct and indirect causes of deforestation are complex and often comprise multiple factors that operate at the same time, e.g., agricultural expansion in combination with wood extraction and expansion of infrastructure^5^. To date, most of these drivers have been determined at a more local scale through case studies or empirical research. With the opening up of the Landsat archive (30 m spatial resolution), Google Earth access to very high-resolution satellite imagery time series (up to 1 m spatial resolution), and improved computing power and storage, the drivers of deforestation have now been mapped globally, covering the years 2001 to 2015^6^. The results indicated that around 50% of deforestation was due to agricultural expansion, with around half of that due to commodity-driven, large-scale agriculture and the rest due to shifting cultivation.

**Fig. 1:**
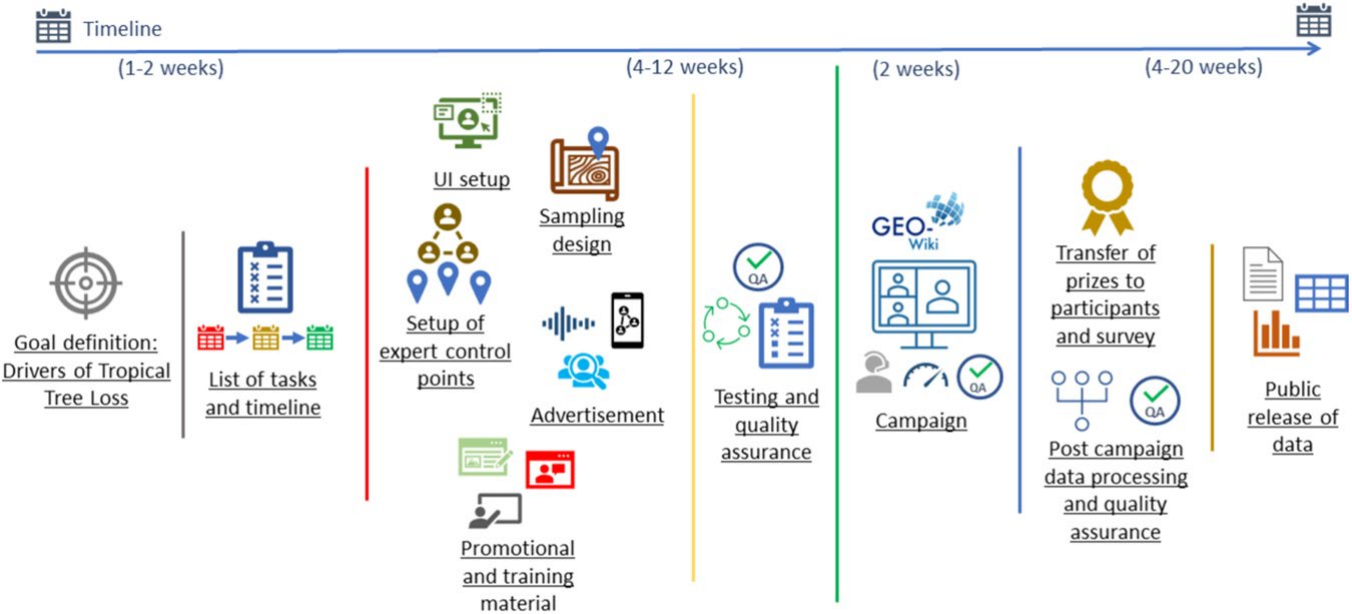
Schema depicting the planning and execution of the ‘Drivers of Tropical Forest Loss’ campaign, from goal definition to final publication of the data.

Determining where commodity-driven deforestation is occurring is required for meeting the targets set out in the European Union’s (EU) Renewable Energy Directive (RED) and the Recast of RED (REDII)^7,8^. When deforestation results from the displacement of food or feed production due to first generation biofuels, this is referred to as indirect land use change (ILUC). High ILUC-risk fuels are defined as biofuels, bioliquids and biomass fuels produced from food and feed crops in which a significant expansion of the production area is on land with high-carbon stocks; according to REDII, these must be reduced to 0% by 2030^8^. Hence, the present data set has been obtained as part of a current review of high ILUC-risk fuels (referred to hereafter as the HILUC project) that builds on a previous study^9^, updating the map of drivers of global forest loss (2001–2015)^6^ for the period 2008 to 2019 for tropical forests. Similar to Curtis *et al*.^6^, very high-resolution satellite imagery was used to visually interpret drivers of tropical forest loss, but here the data were collected through a Geo-Wiki (www.geo-wiki.org) crowdsourcing campaign that ran from the 9th to the 23rd of December, 2020. In total, 58 participants from several countries joined the crowdsourcing campaign, resulting in the collection of 115 K sample locations, each of these visually evaluated by at least 3 different participants, distributed randomly in areas of tree loss in tropical forests since 2008 as sampled from Hansen *et al*.^10^. A general overview of the campaign preparation, the selection of the sample locations, the campaign execution and the post-processing of the data is shown in Fig. 1.

The data and subsequently produced updated maps will be used to inform the current review of high ILUC-risk fuels regarding the proportion of commodity-driven forest loss that can be allocated to specific commodities such as oil palm, maize, etc. The data set can also be used to determine the main causes of forest loss over the last decade.

## Methods

**Fig. 2:**
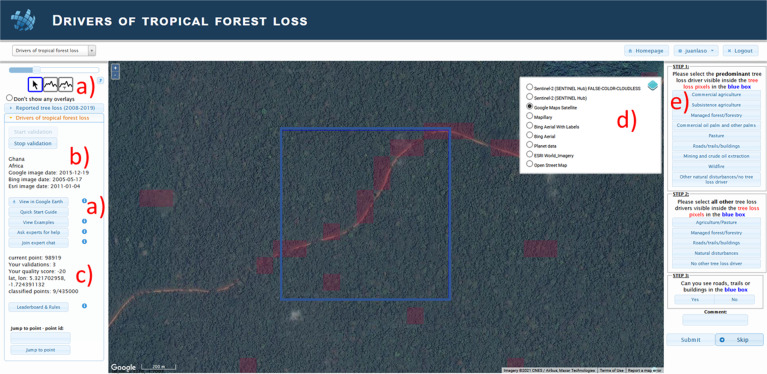
Customized Geo-Wiki interface for the ‘Drivers of Tropical Forest Loss’ crowdsourcing campaign showing: (**a**) Tools available to participants such as the NDVI and Sentinel time-series profiles, visualizing the location on Google Earth and exploring the imagery time-series, reviewing the quick-start guide and exploring examples to identify specific drivers of forest loss as well as contacting IIASA staff via chat or email; (**b**) country and continent of the location as well as dates of the imagery shown; (**c**) campaign statistics; (**d**) available background imagery; and (**e**) tasks to be undertaken by the participants along with buttons to submit or skip the location.

The crowdsourcing campaign was organized as a competition with prizes offered to those who contributed the most, based on a combination of quality and quantity. The Geo-Wiki platform (www.geo-wiki.org), a web platform dedicated to engaging citizens in environmental monitoring, was used as the tool to perform the campaign. A customized user interface was prepared for the campaign (Fig. 2), where participants were shown a random location in the tropics (here broadly defined as the area between 30 degrees of latitude north and south of the equator, i.e., including part of the subtropics), where a blue 1 × 1 km box showed the location to be visually interpreted. The Global Forest Change (GFC) tree loss map (v1.7)^10^ was overlaid on the imagery to show all areas where tree loss was detected at any point between 2008 and 2019. The tree loss area was shaded in red and the map itself was aggregated to 100 m for fast rendering.

The year 2008 was selected as the start date because the RED states that date as the cut-off year for conversion from high-carbon areas, i.e., forest, to other land uses^7^. In order to capture the main drivers of forest loss, but also include potential additional drivers such as the existence of roads as precursors of deforestation, the participants were asked to complete three steps: 1) To select the predominant tree loss driver visible inside the tree loss pixels in the blue box from a list of nine specific drivers; 2) to select all other tree loss drivers visible inside the tree loss pixels in the blue box from a list of five more general drivers, and 3) to mark if roads, trails, or buildings were visible in the blue box. The list of specific and general drivers as well as their definitions is shown in Table 1. The Geo-Wiki interface allowed participants to switch between different background imagery such as ESRI, Google Maps, and Bing Maps as well as Sentinel 2 satellite imagery. The different sources of imagery allowed the participants to see the location at different resolutions and in different periods of time. It also provided participants with information about the current country and the continent as well as the dates of the background imagery. Furthermore, it provided the participants with links for displaying NDVI and Sentinel time series, and to see the location and explore the historical imagery using the Google Earth platform. All these tools were meant to help with easier identification of the forest loss drivers by allowing participants to look at the locations during different times and at different spatial resolutions.

**Table 1:** List and description of the available list of tree loss drivers that participants could select for steps 1 and 2 of the campaign.

Specific tree loss driver (step 1)	Description
Subsistence agriculture	Relatively small fields (typically less than a quarter of the blue box), sometimes without defined borders. Includes agroforestry (combined trees and annual crops) in small fields, shifting cultivation (e.g., abandoned or burnt fields, fallow, bushes). Also includes mixed systems, where small crop fields, small scale pastures and cattle, small patches of palms might be seen, e.g., in some countries in Africa. Time-series helps for identification.
Commercial agriculture	Uniform/monoculture, large fields (typically larger than one quarter of the blue box), but sometimes they can be several small fields together or close by, all with the same land use. Includes permanent (tree-crops/fruit trees) or annual crops, e.g., citrus plantations, olive trees, vineyards, cereals, soybean, sugar cane, banana and cacao plantations. It also includes commercial aquaculture. Excludes oil palm plantations.
Commercial oil palm and other palm plantation	Includes oil palm and other palms such as coconut plantations in large sized fields or several small fields together.
Pasture	Managed grasslands typically with signs of cattle around. May include sparse trees. Ponds of water with roads leading to or criss-crossing trails may be signs of pasture. No obvious agricultural management visible.
Managed forest/forestry	Includes logging, timber plantations, managed forest and replanted forest. This class also includes rubber plantations.
Roads/trails/buildings	Includes any built-up areas and infrastructure, as well as big and small roads or trails that may show human impact or urban expansion.
Mining and crude oil extraction	Includes open mining, small regular oil extraction platforms and dams.
Wildfire	A large (typically larger than the blue box) burnt area with irregular borders.
Other natural disturbances / no tree loss driver	Drought, windthrow (trees uprooted by wind), flooding or no visible tree loss driver.
**General tree loss driver (step 2)**	**Description**
Agriculture/pasture	Includes the categories commercial and subsistence agriculture, pasture but also commercial oil palm and other palm plantations.
Managed forest/forestry	Includes logging, timber plantations, managed forest and replanted forest. This class also includes rubber plantations.
Roads/trails/buildings	Includes the categories roads/trails/buildings and mining and crude oil extraction.
Natural disturbances	Includes the categories wildfire and other natural disturbances.
No other tree loss driver	Select this if you think there are no, or no other, tree loss drivers visible.

The list and descriptions of each were shown in the gallery of drivers (available here: https://application.geo-wiki.org/Application/modules/drivers_forest_change/guide/gallery.pdf) but also in the interface if users hovered over each potential driver.

At the beginning of the campaign, each participant was shown a quick start guide of the interface and the tasks requested. As shown in Fig. 2, this quick start guide could be accessed again at any point during the campaign. Figure 2 also shows that the interface had buttons for four further functions. The first was to see the gallery of examples with access to pre-loaded video-tutorials and examples of images describing each driver of forest loss and how to do visual interpretation and selection of each of these (available at https://application.geo-wiki.org/Application/modules/drivers_forest_change/drivers_forest_change_gallery.html). An illustration of the gallery of examples shown to participants is shown in Figure S1. The second function was to ask experts for help, which automatically sent IIASA experts an email regarding a specific location. The third was to join the expert chat, which led participants to a dedicated chat interface on the Discord messaging platform. Here participants could pose questions and interact with staff and other participants directly. Finally, there was a button to see the leader board as well as the aims, rules and prizes of the campaign (available at https://application.geo-wiki.org/Application/modules/drivers_forest_change/drivers_forest_change.html). When the participants started the campaign, they were shown 10 initial practice locations, where they could try out the user interface (UI) with control points, which showed the participants how to identify the different drivers of forest loss. This set of videos, the images and the training points, together with the gallery of images, were developed to train the participants before and during the campaign.

### Campaign set-up and data quality

As the aim of the campaign was to determine the drivers of tree loss across the tropics, the sample locations were selected from the GFC tree loss layer^10^ for the tropics (between 30 degrees north and south of the equator). No stratification was used since a completely random sample across the tropics was deemed to be the fairest representation of tree loss and their corresponding drivers. The previous map of deforestation drivers^6^ used a 5 K sample of 10 × 10 km grid cells to produce a global map. Here the sample size was largely driven by the estimated capacity of the crowd. Hence, we aimed to validate ca. 150k 1 × 1 km locations across the tropics, which is a considerably larger sample size than that of Curtis *et al*.^6^. In order to reduce noise, the GFC tree loss layer^10^ was first aggregated to a 100 m resolution from the original 30 m, and 150 K centroids were then randomly selected. From these, a sub sample of 5000 random locations were selected for visual interpretation by six IIASA experts (with backgrounds in remote sensing, agronomy, forestry and geography). Due to time constraints, only 2001 locations were evaluated by at least three different experts. In these locations, agreement was discussed and once a consensus was reached, these locations became the final control or expert data set. The control locations were then used to produce quality scores for each participant as the campaign progressed in order to rank them and determine the final prize winners. The list of prizes offered to the top 30 participants is shown in Table S1 in the Supplementary Information (SI), and a list and rank of motivations mentioned by the participants is shown on Figure S2 in the SI.

The control locations were randomly shown to the participants at a ratio of approximately 2 control locations to every 20 non-control locations visited. If the participants correctly selected the predominant tree loss driver (in step 1), they were awarded 20 points; if they selected the wrong answer, they lost 15 points. If participants confused pasture and commercial agriculture or wildfire with other natural disturbances, they lost only 10 points instead of 15. Furthermore, they could win 8 additional points by selecting the correct secondary drivers in step 2. If a mixture of correct and incorrect answers were provided in step 2, the participants gained 2 points for every correct choice and lost 2 points for every incorrect one, with a minimum gain/loss of 0 points. Finally, participants could earn 2 additional points by correctly reporting the existence of roads, trails or buildings in step 3. The scoring system was based on previous Geo-Wiki campaign experiences and aimed to promote focus on the primary driver selection. The points were used to produce a leader board with the total number of points by participant. Additionally, a relative quality score (RQS) was derived from the score received by the users and the potential score that could have been obtained if all control points were correctly interpreted. This is shown in Eq. 1.

1 $${\rm{RQS}}=(({{\rm{NCP}}}^{\ast }15+{\rm{SumScore}})/{\rm{NCP}})/45$$

where RQS ranges between 0 and 1, NCP is the number of control points visited and SumScore is the number of points obtained.

The RQS was crucial in understanding how each participant performed in terms of the quality of their visual interpretations, as this was independent of the number of locations interpreted. Once the campaign ended, an average RQS was used as a minimum criterion for participants to receive a prize, independent of where they were located on the leader board. Additionally, all users who submitted a substantial number of interpretations, i.e., more than 1000 with the minimum required RQS, were invited to become co-authors of the current manuscript, independent of whether they received a monetary prize or not. All these co-authors additionally contributed to the editing and revision of this manuscript. Furthermore, future users of the data set could use the RQS as a key data quality indicator.

After the campaign, the data post-processing included eliminating interpretations made by users who broke any of the competition rules. Additionally, during the campaign, some users communicated with IIASA staff using the “Ask Experts” button and pointed out that some control points were mistaken. Consequently, the corresponding points lost were added to the final score of those participants where the correction was made. A total of 18742 validations from 1 participant were removed before the end of the campaign and the user was disqualified since their account was deemed to be shared across several people and computers, which was not allowed. Validations from another user (38,502 out of 40,828) were also removed due to inconsistencies but the user remained in the competition. Before the prizes were awarded to the top 30 users, a questionnaire was administered to all users to gather information about participant characteristics and gauge their motivations. Participation was mandatory for the top 30 users. A summary of the participant backgrounds is provided in Figure S3 in the SI.

## Data Records

The data records are accessible for download from the permanent DARE repository hosted by the International Institute for Applied Systems Analysis (IIASA) (https://dare.iiasa.ac.at/122/)^11^. These consist of two files, one containing the full data set (ILUC_DARE_campaign.csv, n = 1,158,021) and the second one containing the control or expert data (ILUC_DARE_controls.csv, n = 6,157, corresponding to 2001 locations). Note that each location contains at least 3 rows, i.e., one answer per step, with step 2 containing sometimes more than one answer. Table 2 shows the format and information contained in the data provided.

**Table 2:** Variables included in the data records, format, and examples of the data.

Campaign data file (n = 1,158,021)			
Variable	Short description	Type of variable	Example value
userid	Unique user identifier	Numeric	20326
submissionid	Unique submission identifier	Numeric	2031536
timestamp	Date and time when the submission was done	Date-time	09DEC20:13:26:38
sampleid	Unique sample (location) identifier	Numeric	1738073
skip	Whether the location was skipped (1) or not (0)	Numeric	0
viewed_ge	Whether the participant clicked on the “View in Google Earth” (1) button or not (0)	Text	FALSE
samplegroupid	Whether the location visited corresponds to a control point (230) or not (229)	Numeric	229
used_basemap	The basemap that was active when the submission was done: 1 = Google Maps, 2 = Bing Maps, 3 = ESRI imagery	Numeric	1
submission_itemid	Unique identifier for each answer provided by an individual participant	Numeric	58506737
legendid	Unique numeric identifier for every step of the campaign: Step1 = 298, Step2 = 299, Step 3 = 301.	Numeric	299
step	Step (question) being answered by the participant	Text	step 1
name	Full question (step) being answered by the participant	Text	Please select all other tree loss drivers visible inside the tree loss pixels in the blue box
answer	Answer provided to the question (step) being asked. Step 2 can contain multiple answers	Text	Agriculture/Pasture
comm	Whether the participant provided any additional comment (1) or not (0)	Numeric	1
comments	The comment provided by the participant	Text	shifting cultivation
score_ctrl	Score received by the participant if the location validated was a control location. For non-control locations, missing values are shown (range: −15 to 30).	Numeric	20
qual_score	The total quality score of the participant at the end of the campaign	Numeric	36304
rank_ini	Participant ranking at the end of the campaign	Numeric	5
rqs	Relative quality score at the end of the campaign	Numeric	0.65
addedpts	20000 points added to the quality score of those participants that had over 0.8 rqs on 18^th^ December, 5 pm	Numeric	20000
newqs	Final quality score after addedpts	Numeric	56304
rank_final	Final ranking after addedpts	Numeric	3
x	Geographical coordinate for the centroid of a given location – Longitude	Numeric	97.9239267
y	Geographical coordinate for the centroid of a given location - Latitude	Numeric	26.25604234
**Control data file (n** = **6,157)**			
sampleid	Unique sample (location) identifier	Numeric	1738073
legendid	Unique numeric identifier for every step of the campaign: Step1 = 298, Step2 = 299, Step 3 = 301.	Numeric	299
step	Step (question) being answered by the experts reviewing the control location	Text	step 1
name	Full question (step) being answered by the experts reviewing the control location	Text	Please select all other tree loss drivers visible inside the tree loss pixels in the blue box
answer	Answer provided to the question (step) being asked. Step 2 can contain multiple answers	Text	Agriculture/Pasture
x	Geographical coordinate for the centroid of a given location – Longitude	Numeric	97.9239267
y	Geographical coordinate for the centroid of a given location - Latitude	Numeric	26.25604234

## Technical Validation

**Fig. 3:**
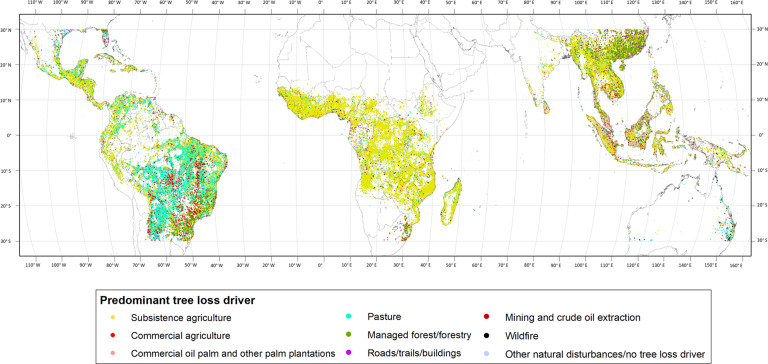
Example of the mapped tropical drivers of forest loss, where the centroids of locations validated three times by participants with an RQS higher than 0.8 are shown with the predominant tree loss driver where at least two out of three participants agreed.

As an example of the data described here, Fig. 3 shows the predominant tree loss driver allocated to each of the locations where 3 visual interpretations by different participants were done. The data shown in the map comes from participants with an RQS higher than 0.8.

**Fig. 4:**
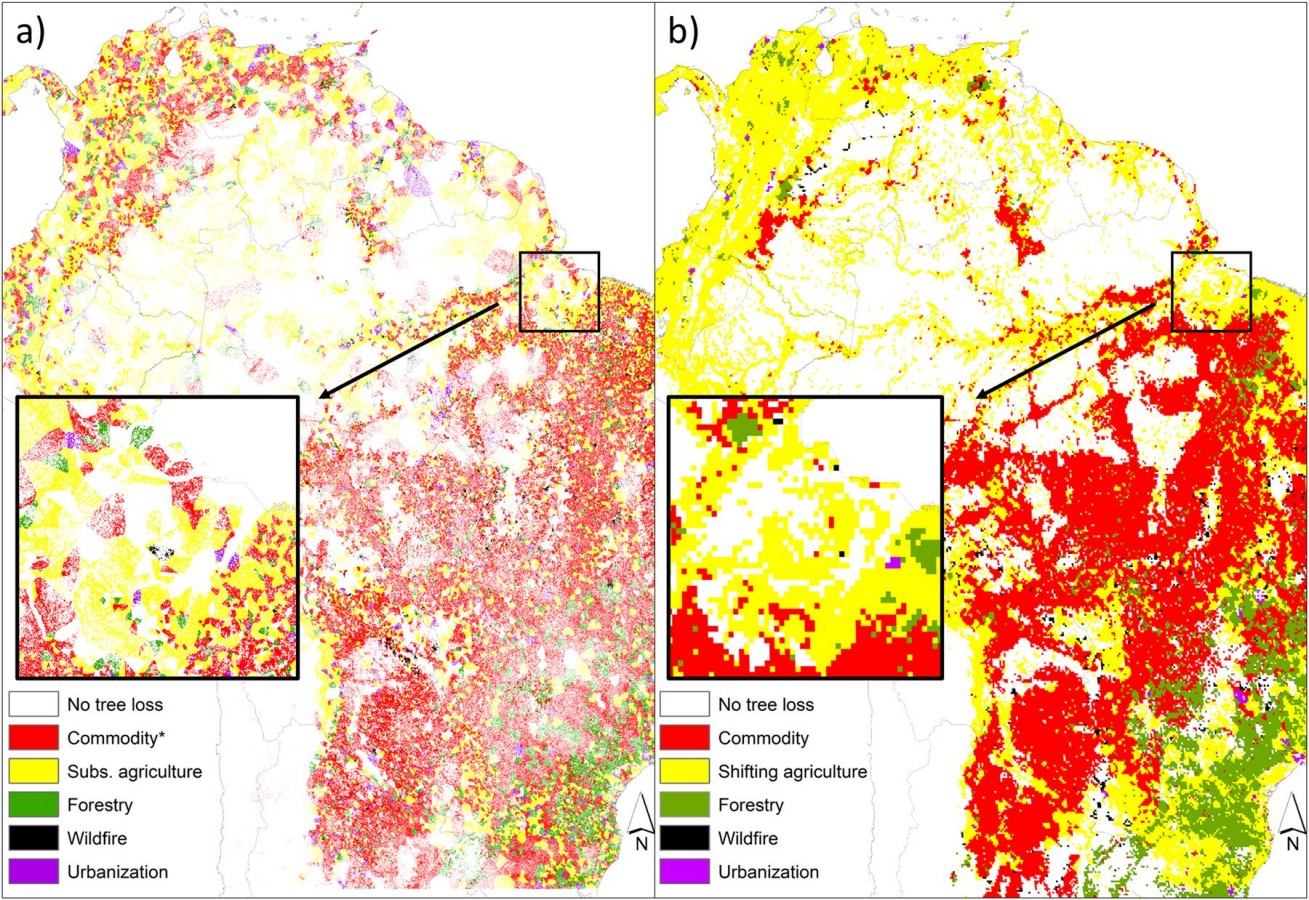
Comparison of a map layer produced with the drivers of tropical forest loss data set against the Curtis *et al*.^6^ drivers of forest loss layer in South America. Here we contrast (**a**) an initial tessellation map produced from the data set, where the Hansen *et al*.^10^ 100 m aggregated tree loss pixels were allocated the predominant tree loss driver in the area, and (**b**) the Curtis *et al*.^6^ drivers of forest loss layer (adapted from the original data source). *In the tessellation map, the class Commodity contains commercial agriculture, commercial oil palm and other palm plantations, pasture, and mining and crude oil extraction. All classes are shown in similar palette colours as those from the Curtis *et al*.^6^ layer to match their drivers of forest loss definition and to provide an easier visual comparison of both maps. The inset areas highlight the improved resolution of the new data.

An additional example (Fig. 4) shows an initial test map produced using the data shown in Fig. 3. Here the Hansen *et al*.^10^ 100 m aggregated pixels were allocated to the predominant tree loss driver that was in the vicinity. Figure 4 compares this map with the Curtis *et al*.^6^ layer to give an idea of the finer resolution data produced in the campaign but also general agreement in forest drivers and spatial patterns.

## Usage Notes

The data will be used to inform the current review of high ILUC-risk fuels regarding the proportion of commodity-driven forest loss that can be allocated to specific commodities such as oil palm, maize, etc. However, the data set can be used more generally to determine the main causes of forest loss over the last decade. Due to its higher resolution, the data set provided constitutes a rich source of information for policy makers and practitioners. The user information provided for each record, especially the RQS, can be employed to sort through the data and obtain the highest quality information.

## Supplementary information

Supplementary Information

## Data Availability

No custom code was used to generate or process the data described in the manuscript.
