# Supplementary material for: Drivers of tropical forest loss between 2008 and 2019
Source: Sci Data. 2022 Apr 1;9:146. doi: 10.1038/s41597-022-01227-3 (PMC8976004; doi:10.1038/s41597-022-01227-3)
Supplement: Supplementary file 1 — Supplementary Information [file 41597_2022_1227_MOESM1_ESM.pdf]

Supplementary Information for Drivers of tropical forest loss between 2008 and 2019

Authors

Juan Carlos Laso Bayas[1], Linda See[1], Ivelina Georgieva[1], Dmitry Schepashchenko[1,2], Olga Danylo[1], Martina Dürauer[1], Hedwig Bartl[1], Florian Hofhansl[1], Myroslava Lesiv[1], Roman Zadorozhniuk[3], Maksym Burianchuk[3], Flavius Sirbu[4], Brigitte Magori[4], Kateryna Blyshchyk[4], Volodymyr Blyshchyk[4], Ahmed Harb Rabia[5], Chandra Kant Pawe[6], Yuan-Fong Su[7], Merajuddin Ahmed[8], Kripal Panging[9], Oleksandr Melnyk[3], Olesia Vasylyshyn[3], Roman Vasylyshyn[3], Andrii Bilous[3], Svitlana Bilous[3], Krishna Das[6], Reinhard Prestele[10], Ana Pérez-Hoyos[11], Khangsembou Bungnamei[9], Andrii Lashchenko[3], Maryna Lakyda[3], Ivan Lakyda[3], Oleksandr Serediuk[3], Galyna Domashovets[3], Yuriy Yurchuk[3], Michèle Koper[12], and Steffen Fritz[1]

Affiliations

1. International Institute for Applied Systems Analysis (IIASA), Laxenburg, Austria
2. Center of Forest Ecology and Productivity of the Russian Academy of Sciences, Profsoyuznaya 84/32/14, Moscow 117997, Russia
3. National University of Life and Environmental Sciences of Ukraine (NULESU), Heroiv Oborony 15, Kyiv, Ukraine, 03041
4. West University of Timisoara, Bulevardul Vasile Parvan 4, Timisoara 300323, Romania
5. Damanhour University, Faculty of Agriculture, Natural Resources & Agricultural Engineering Department, El-abaadya Campus, Damanhour 22516, El-Behera, Egypt
6. Department of Geography, Pragjyotish College, Guwahati-09, Assam, India
7. Department of Harbor and River Engineering, National Taiwan Ocean University, No. 2, Beining Rd., Zhongzheng Dist., Keelung City 202, Taiwan (R.O.C.)
8. Northeast centre for Equity Action on Integrated Development (NEAID), NEAID (Registered Office), G.R.C Road, Noonmati, Guwahati, Kamrup(M), Assam, India- 781020
9. Department of Geography, Gauhati University, Jalukbari, Guwahati, Assam 781014, India
10. Institute of Meteorology and Climate Research - Atmospheric Environmental Research (IMK-IFU), Karlsruhe Institute of Technology (KIT), Kreuzeckbahnstraße 19, 82467 Garmisch-Partenkirchen, Germany
11. Joint Research Center of the European Union (JRC), Via Enrico Fermi, 2749, I-21027 Ispra, Italy
12. Guidehouse, Stadsplateau 15, 3521, AZ-Utrecht, The Netherlands

Table of Contents

Figure S1. Example of the training materials shown in the gallery of examples of the 'Drivers of Tropical Forest Loss' campaign, where pasture is the predominant driver and Sentinel time-series are also shown as a useful tool for driver identification. Additional video tutorials available to the participants can be seen at https://www.youtube.com/playlist?list=PLQarHncFYDMWBQI4HvGWVOIQeqNQTP9mj 3

Table S1. List of prizes offered to the top 30 participants of the 'Drivers of Tropical Forest Loss' crowdsourcing campaign. 4

Figure S2. Rank of motivations as mentioned by participants of the 'Drivers of Tropical Forest Loss' Geo-Wiki campaign that participated in the post-campaign survey (n=42) 5

Figure S3. Characteristics of the participants of the 'Drivers of Tropical Forest Loss' campaign. The information shown comes from those participants that provided voluntary feedback on a survey at the end of the campaign (n=42). 6

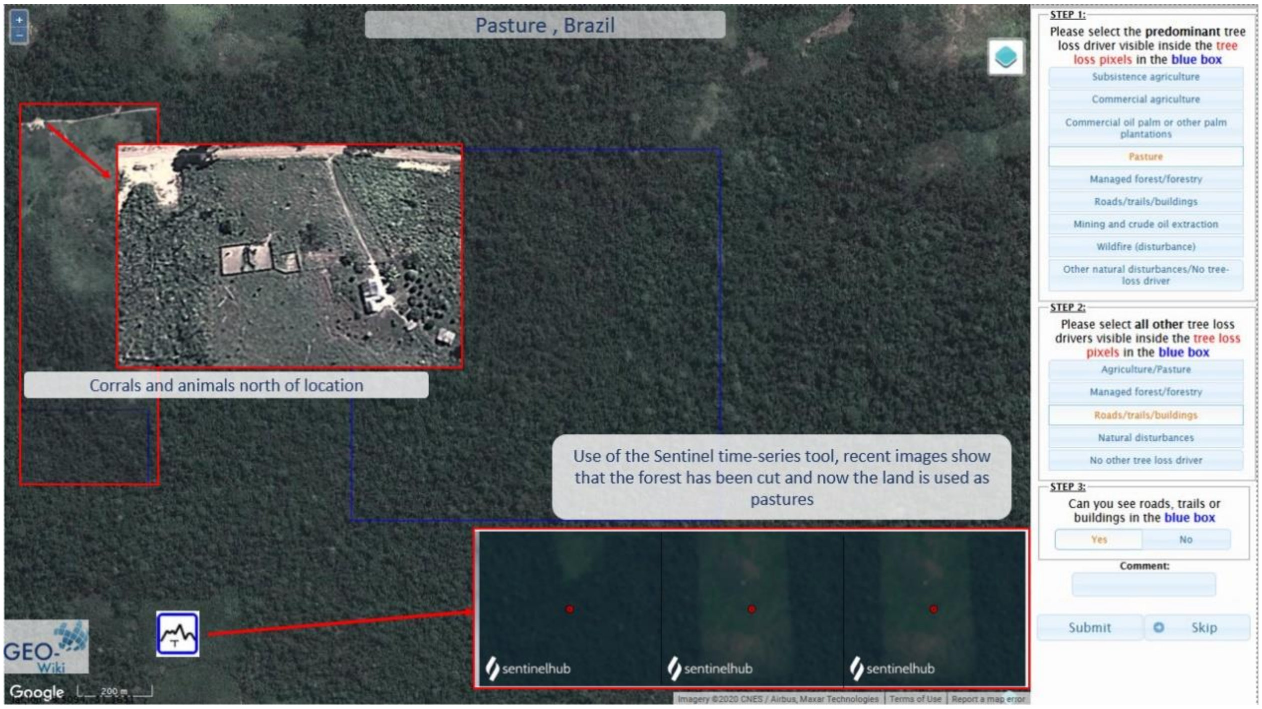

Figure S1. Example of the training materials shown in the gallery of examples of the 'Drivers of Tropical Forest Loss' campaign, where pasture is the predominant driver and Sentinel time-series are also shown as a useful tool for driver identification. Additional video tutorials available to the participants can be seen at https://www.youtube.com/playlist?list=PLQarHncFYDMWBQI4HvGWVOIQeqNQTP9mj

Table S1. List of prizes offered to the top 30 participants of the 'Drivers of Tropical Forest Loss' crowdsourcing campaign.

Ranking	Prize (€)	Ranking	Prize (€)	Ranking	Prize (€)
**1**	300	**11**	200	**21**	75
**2**	275	**12**	200	**22**	75
**3**	275	**13**	175	**23**	75
**4**	275	**14**	175	**24**	75
**5**	275	**15**	150	**25**	75
**6**	250	**16**	150	**26**	75
**7**	250	**17**	125	**27**	75
**8**	250	**18**	125	**28**	75
**9**	250	**19**	125	**29**	75
**10**	250	**20**	125	**30**	75

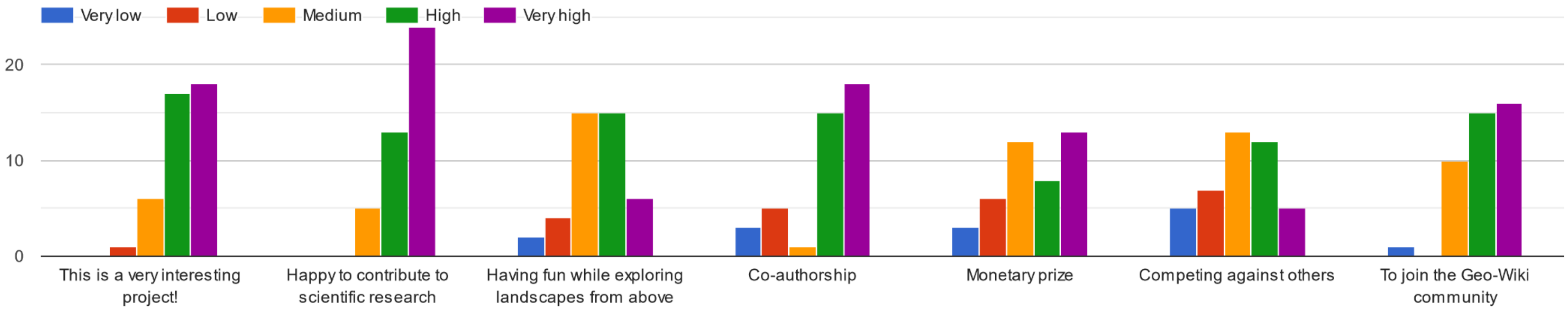

Figure S2. Rank of motivations as mentioned by participants of the 'Drivers of Tropical Forest Loss' Geo-Wiki campaign that participated in the post-campaign survey (n=42)

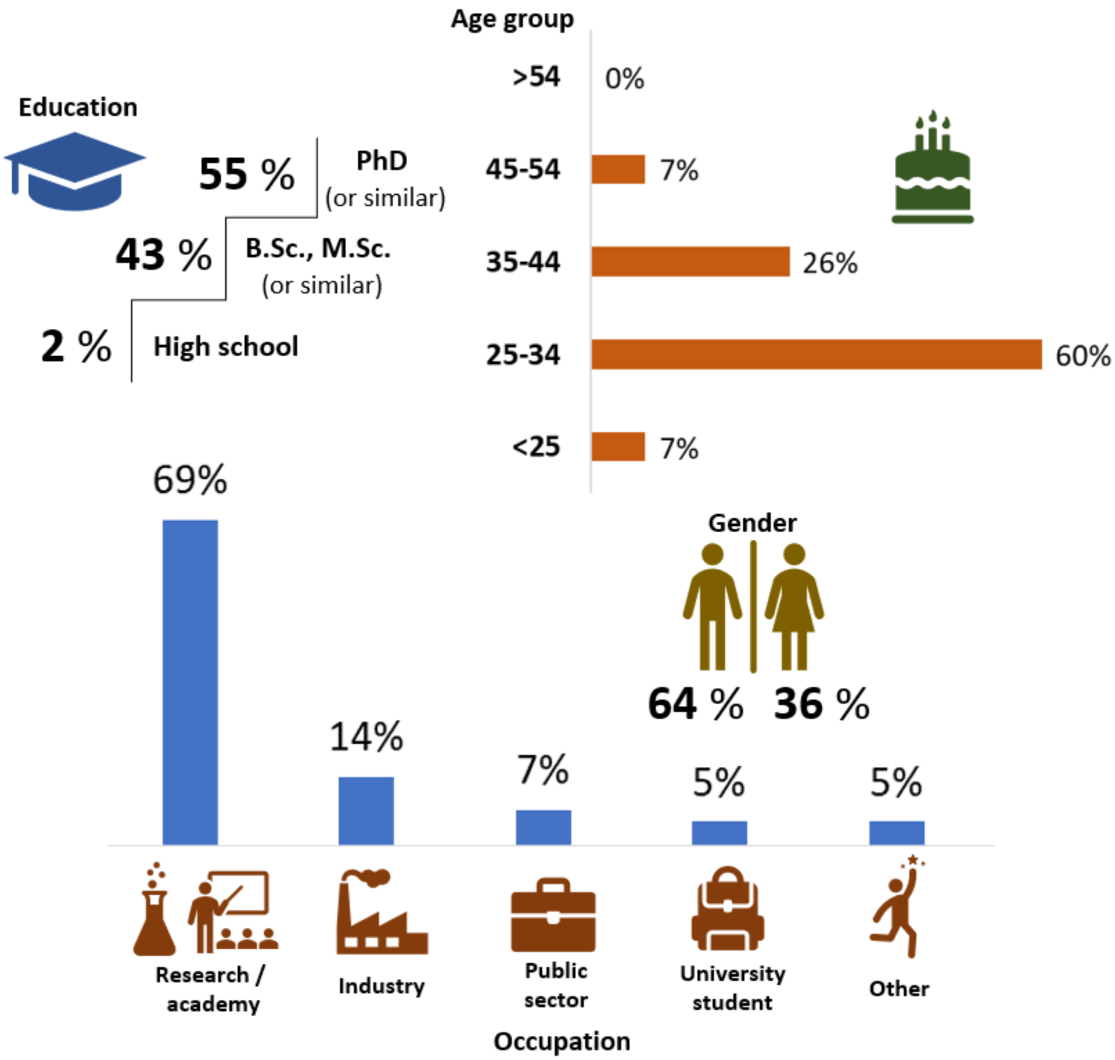

Figure S3. Characteristics of the participants of the 'Drivers of Tropical Forest Loss' campaign. The information shown comes from those participants that provided voluntary feedback on a survey at the end of the campaign (n=42).
